# Dysnatremia is associated with increased risk of all-cause mortality within 365 days post-discharge in patients with atrial fibrillation without heart failure: A prospective cohort study

**DOI:** 10.3389/fcvm.2022.963103

**Published:** 2022-10-12

**Authors:** Yan Zhou, Dong Lin, Shiwan Wu, Jiaxin Xiao, Min Yu, Zhongbo Xiao, Muli Wu, Zhisheng Chen, Cuihong Tian, Rongbing Chen, Yequn Chen, Xuerui Tan

**Affiliations:** ^1^Department of Cardiology, First Affiliated Hospital of Shantou University Medical College, Shantou, China; ^2^School of Medical and Health Sciences, Edith Cowan University, Perth, WA, Australia; ^3^Clinical Research Center, First Affiliated Hospital of Shantou University Medical College, Shantou, China

**Keywords:** atrial fibrillation, hyponatremia, hypernatremia, mortality, cohort study

## Abstract

**Aim:**

The aim of this study is to evaluate the association between serum sodium concentrations at hospital admission and all-cause mortality within 365 days post-discharge in patients with atrial fibrillation (AF) without heart failure (HF).

**Methods:**

The prospective cohort study enrolled 1,446 patients with AF without HF between November 2018 and October 2020. A follow-up was performed 30, 90, 180, and 365 days after enrollment through outpatient visits or telephone interviews. All-cause mortality was estimated in three groups according to serum sodium concentrations: hyponatremia (< 135 mmol/L), normonatremia (135–145 mmol/L), and hypernatremia (> 145 mmol/L). We estimated the risk of all-cause mortalities using univariable and multivariable Cox proportional hazards models with normonatremia as the reference.

**Results:**

The all-cause mortalities of hyponatremia, normonatremia, and hypernatremia were 20.6, 9.4, and 33.3% within 365 days post-discharge, respectively. In the univariable analysis, hyponatremia (HR: 2.19, CI 1.5–3.2) and hypernatremia (HR: 4.03, CI 2.32–7.02) increased the risk of all-cause mortality. The HRs for hyponatremia and hypernatremia were 1.55 (CI 1.05–2.28) and 2.55 (CI 1.45–4.46) after adjustment for age, diabetes mellitus, loop diuretics, antisterone, antiplatelet drugs, and anticoagulants in the patients with AF without HF. The association between serum sodium concentrations and the HRs of all-cause mortality was U-shaped.

**Conclusion:**

Dysnatremia at hospital admission was an independent factor for all-cause mortality in patients with AF without HF within 365 days post-discharge.

## Introduction

Atrial fibrillation (AF), which is the most common form of cardiac arrhythmia and is triggered in lifestyle-related conditions such as diabetes mellitus (DM) and stress (1–3), accounts for substantial morbidity and mortality (4–6), and the incidence of AF is expected to at least double by 2050 (7). AF is also associated with a nearly 5-fold increase in the risk of ischemic stroke (8) and heart failure (HF) (9, 10), and an increased financial burden (11, 12). Serum sodium is the main component of human plasma responsible for osmotic pressure and the main participant in heart electrical activity. Dysnatremia, including hyponatremia and hypernatremia, is very common in hospitalized patients (13, 14) and complications of acute diseases, and results from interventions during patient treatment (15, 16) or exists as a comorbid condition (17). Previous studies have shown that abnormal serum sodium concentrations are independent factors for poor prognosis of internal medicine patients (18–20) and patients with cardiac diseases (14, 21, 22). Studies show that patients with HF with AF frequently present with hyponatremia (23, 24), which is associated with high mortality (25). The incidence of abnormal sodium levels in non-HF patients is lower compared with patients with HF. However, it is unknown whether abnormal serum sodium levels play a role in the pathophysiology of and can be predictors of long-term poor prognosis in patients with AF without HF. In this study, we determine the association between serum sodium concentrations at the time of hospital admission and all-cause mortality over the 365 days following discharge in patients with AF without HF.

## Methods

### Study cohort

The data used were sub-data obtained from a single-center prospective observational cohort study in which 2011 patients with AF were enrolled from November 2018 to October 2020 at the First Affiliated Hospital of Shantou University Medical College in Shantou, China. Briefly, AF was diagnosed by a physician according to heart rhythm by ECG. The diagnostic criteria for ECG were (1) absolutely irregular RR intervals, (2) no discernible or distinct P waves, and (3) an episode lasting at least 30 s. The inclusion criteria were diagnosis of AF and age >18 years old. The exclusion criteria were pregnancy, death in the hospital, HF, and refusal of follow-up. A follow-up was performed through a clinic visit or telephone interview 30, 90, 180, and 365 days after discharge. The primary endpoint was all-cause death in participants after discharge from the hospital. The patients were grouped based on admission serum sodium levels defined as follows: hyponatremia (< 135 mmol/L), normonatremia (135–145 mmol/L), and hypernatremia (> 145 mmol/L).

### Data collection

All the patients with AF received a systemic clinical evaluation at the beginning of hospitalization. Data were collected, including age, sex, comorbidities, medication, and serum sodium concentrations, from patient hospital records. The serum sodium concentrations used in the analysis were obtained within the first 24 h of admission and measured by the clinical laboratory of the First Affiliated Hospital of Shantou University Medical College (instruments: Beckman Coulter AU5800 automatic biochemical analyzer, method: direct ion-selective electrodes). Hypertension was defined as blood pressure over 140 and/or 90 mmHg measured at least two different times over 4 h or taking an antihypertensive medication. DM was defined as fasting blood glucose of ≥7 mmol/L, random blood glucose ≥11.1 mmol/L, or taking an antidiabetic medication. Coronary artery disease was diagnosed as having more than one coronary stenosis of >50% by coronary artery angiography or having a history of coronary artery disease. HF was diagnosed by a senior clinician according to an ejection fraction lower than 50% by echocardiography combined with symptoms (such as shortness of breath) and physical signs (such as edema) or elevated N-terminal pro-B-type natriuretic peptide and evidence of diastolic dysfunction (26). Medicines that the patients were taking contained amiodarone, loop diuretics, antisterone, angiotensin-converting enzyme inhibitors/angiotensin II receptor blockers, antiplatelet drugs, anticoagulants, and beta-blockers.

This study complied with the principles of the Declaration of Helsinki and was approved by the Ethics Committee of the First Affiliated Hospital of Shantou University Medical College.

### Statistical analyses

Non-normal variables (age, median survival time) are presented as the median and interquartile range, and a Kruskal-Wallis rank-sum test was conducted to evaluate differences. Categorical data were determined as counts or percentages, and differences were evaluated by a Pearson χ^2^ test. Kaplan-Meier survival curves were plotted for three sodium levels to illustrate survival. In order to investigate the association between dysnatremia and all-cause mortality within 30–365 days, we used univariable and multivariable Cox proportional hazards models. The relationship between serum sodium concentration and the unadjusted hazard ratio (HR) of mortality was assessed using a restricted cubic spline curve based on Cox proportional hazards models (27, 28). We considered a two-sided *p*-value below 0.05 to be statistically significant and below 0.01 to be highly statistically significant. The statistical analyses were performed using SPSS 23.0 for Windows (version 23.0; IBM Corp., Armonk, NY) and the R (version 4.0.2; R Foundation for Statistical Computing, Vienna, Austria) software.

## Results

**Figure 1:**
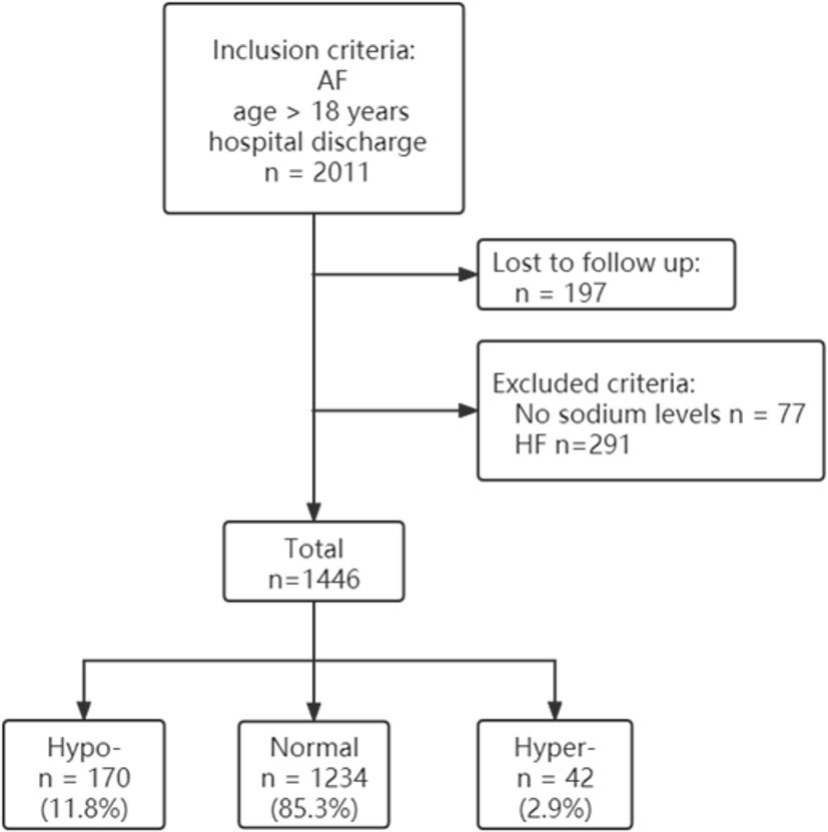
Participant flow diagram. AF, atrial fibrillation; HF, heart failure; Hypo, hyponatremia; Normal, normonatremia; Hyper, hypernatremia.

**Figure 2:**
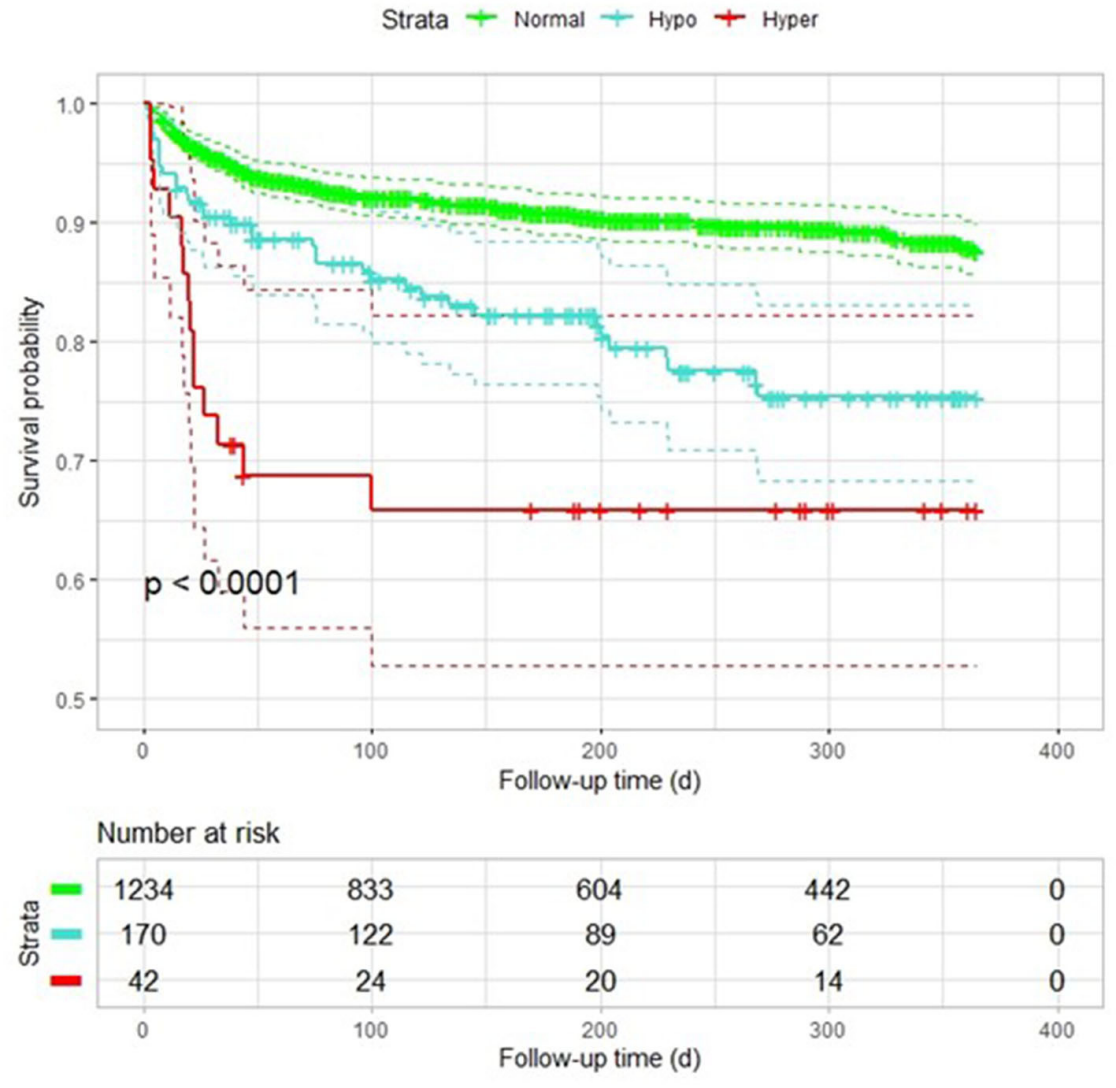
Kaplan–Meier plot of survival probability among patients with AF with different sodium levels. Hypo, hyponatremia; Normal, normonatremia; Hyper, hypernatremia.

Data were selected according to the flow chart shown in Figure 1. Among 2,011 participants, 197 were excluded because of lack of follow-up data, 77 patients were excluded because of missing baseline serum sodium concentrations, and 291 were excluded because of history of heart failure. The final analysis included 1,446 patients with diagnosed AF but without HF. The baseline characteristics of the study population according to each sodium group are shown in Table 1. The prevalence rate of hypo- and hypernatremia was 11.8 and 2.9%, respectively. Of the 1,446 patients, 165 (11.4%) died between 30 and 180 days. This population was characterized by advanced age, with the hyponatremic and hypernatremic patients being older than the normonatremic patients (Table 1). The all-cause mortality in the three groups from hyponatremia, normonatremia, and hypernatremia was 35 (20.6%), 116 (9.4%), and 14 (33.3%), respectively. Significant differences among the three groups were observed for age and prevalence of DM, as well as taking loop diuretics, antisterone, antiplatelet drugs, and anticoagulants. The association between all-cause mortality and serum sodium concentrations (hyponatremia, normonatremia, and hypernatremia) is illustrated as Kaplan–Meier survival curves (Figure 2).

**Table 1:** Baseline characteristics of the study participants.

**Parameters**	**Total**	**Hyponatremia**	**Normonatremia**	**Hypernatremia**	***p*-value**
	***n* = 1,446**	***n* = 170**	***n* = 1234**	***n* = 42**	
Age (range), years	70 (62–77)	74 (65–81)	69 (61–77)	70 (65–77)	<0.001
Males, *n* (%)	836 (57.8%)	87 (51.2%)	725 (58.8%)	24 (57.1%)	0.172
DM, *n* (%)	379 (26.2%)	65 (38.2%)	301 (24.4%)	13 (31.0%)	<0.001
Hypertension, *n* (%)	885 (61.2%)	102 (60.0%)	756 (61.3%)	27 (64.3%)	0.872
CAD, *n* (%)	621 (42.9%)	63 (37.1%)	543 (44.0%)	15 (35.7%)	0.145
Amiodarone, *n* (%)	241 (16.7%)	24 (14.1%)	208 (14.1%)	9 (21.4%)	0.469
Loop diuretics, *n* (%)	851 (58.9%)	142 (83.5%)	677 (54.9%)	32 (76.2%)	<0.001
Antisterone, *n* (%)	609 (42.1%)	110 (64.8%)	482 (39.1%)	17 (40.5%)	<0.001
ACEI/ARB, *n* (%)	440 (30.4%)	54 (31.8%)	375 (30.4%)	11 (26.2%)	0.779
Antiplatelet drugs, *n* (%)	666 (46.1%)	61 (35.9%)	581 (47.1%)	24 (57.1%)	0.008
Anticoagulants, *n* (%)	992 (68.6%)	104 (61.2%)	866 (70.2%)	22 (52.4%)	0.004
Beta-blockers, *n* (%)	907 (62.7%)	97 (57.1%)	784 (63.5%)	26 (61.9.2%)	0.260
Median survival time (range), days	188 (46–359)	201 (60–356)	187 (46–361)	182 (22–346)	0.868
Deaths, *n* (%)	165 (11.4%)	35 (20.6%)	116 (9.4%)	14 (33.3%)	<0.001

*DM*, diabetes mellitus; *CAD*, coronary artery disease; *ACEI*, angiotensin-converting enzyme inhibitors; *ARB*, angiotensin II receptor blockers.

The results of the multivariable-adjusted analysis with normal sodium are shown in Table 2. The all-cause mortality rates were higher in patients with hyponatremia (HR: 2.19, CI: 1.5–3.2) and hypernatremia (HR: 4.03, CI: 2.32–7.02) than in normonatremia in the univariate Cox hazards analyses. In model 1, the HR for hyponatremia was 1.88 (CI: 1.29–2.76), and the HR for hypernatremia was 3.85 (CI: 2.21–6.71) after adjustment for age. In model 2, after adding DM to model 1, the HR for hyponatremia and hypernatremia was 1.86 (CI: 1.27–2.73) and 3.86 (CI: 2.21–6.71), respectively. In model 3, the mortality rate remained significantly high in patients with hyponatremia (HR: 1.55, CI: 1.05–2.28) and hypernatremia (HR: 2.55, CI: 1.45–4.46) after adjustment for age, prevalence of DM, loop diuretics, antisterone, antiplatelet drugs, and anticoagulants.

**Table 2:** Association of sodium with risk of all-cause mortality in patients with AF.

**Model**	**Normonatremia**	**Hyponatremia HR (95% CI)**	***p*-value**	**Hypernatremia HR (95% CI)**	***p*-value**
Unadjusted	Reference	2.19 (1.50–3.20)	<0.001	4.03 (2.32–7.02)	<0.001
Model 1		1.88 (1.29–2.76)	0.001	3.85 (2.21–6.71)	<0.001
Model 2		1.86 (1.27–2.73)	0.002	3.86 (2.21–6.71)	<0.001
Model 3		1.55 (1.05–2.28)	0.029	2.55 (1.45–4.46)	0.001

Values are based on multivariate Cox proportional hazards models. Model 1, adjusted for age; Model 2, Model 1 + DM; Model 3, model 2+ loop diuretics + antisterone + antiplatelet drugs +anticoagulants. *DM*, diabetes mellitus; *HR*, hazard ratio; *95% CI*, 95% confidence interval.

**Figure 3:**
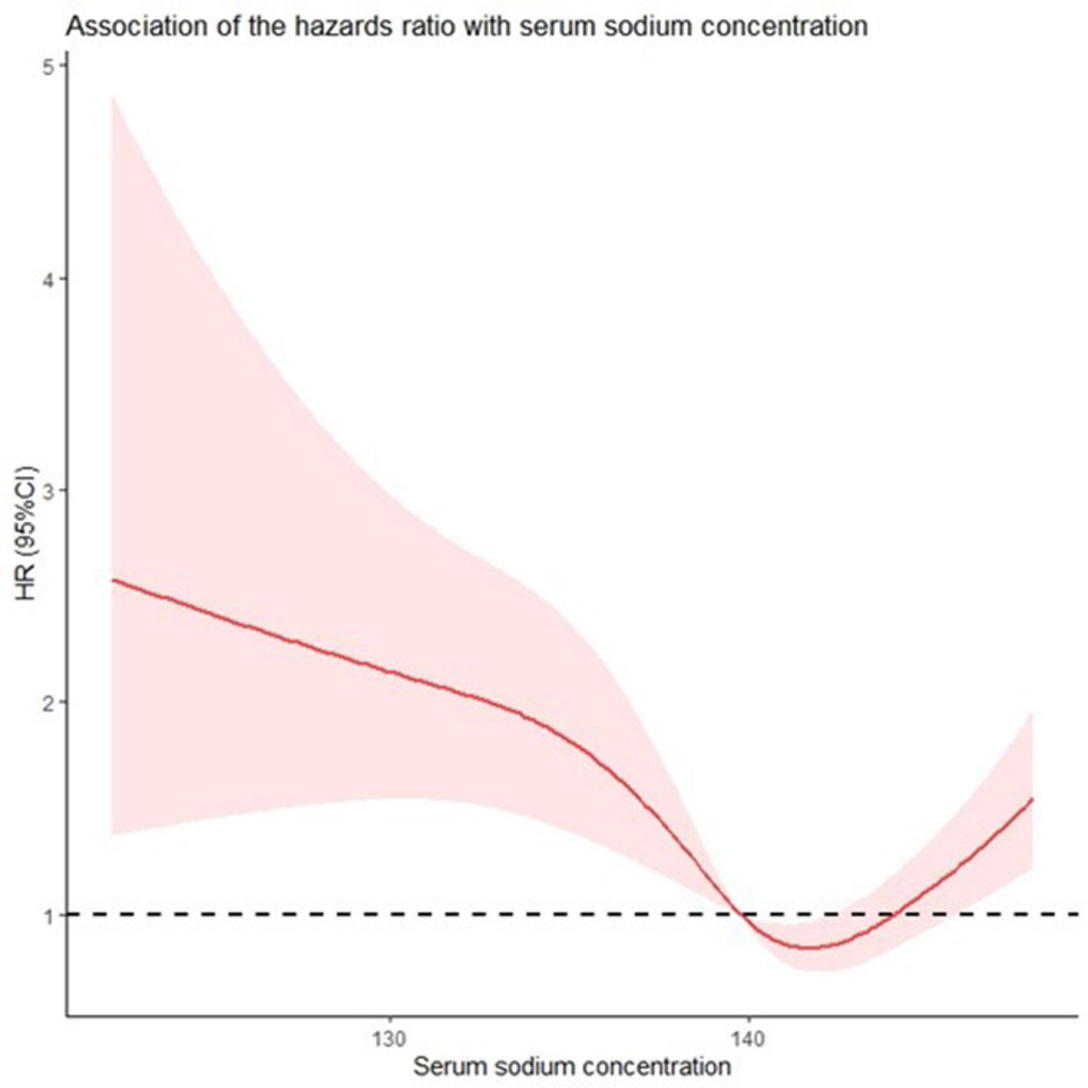
Both hypo- and hypernatremia affect the all-cause mortality of patients with atrial fibrillation without heart failure in a serum sodium concentration-dependent manner. Restricted cubic spline curve (RCS) showing the unadjusted hazard ratios for all-cause mortality as a function of sodium concentration according to univariate Cox proportional hazards analysis.

The relationship between the unadjusted HRs of post-discharge mortality in patients with AF without HF and serum sodium levels was U-shaped (Figure 3). The spline curve showed that both high and low sodium levels increased the risk for all-cause post-discharge mortality in patients with AF with no HF within 365 days.

## Discussion

Our study shows that the dysnatremia at the time of hospital admission in the patients with AF without HF is highly associated with increased risk of all-cause mortality during the 365-day period following discharge. This relationship remains after adjustment for age, comorbidities, and medication. Furthermore, the prognostic relationship between serum sodium concentrations and the unadjusted HRs for all-cause post-discharge mortality exhibits a U-shaped curve, as opposed to a nonlinear association, with a higher risk at both ends of the sodium concentration distribution. It is noteworthy that both hyponatremia and hypernatremia are the risk factors for mortality in the patients with AF without HF.

Limited studies have reported that hyponatremia increased the risk of mortality in patient with AF, mainly in patients with HF. Studies showed that hyponatremia was more common in HF with reduced ejection fraction combined with AF (23, 29). In a prospective multicenter pilot survey that included 215 participants with AF and HF, Ozierański et al. showed that the incidence of hyponatremia was 19.1%, and that hyponatremia at hospital admission was a risk for mortality in patients with AF who have had HF for 12 months (25). In our study, the prevalence of hyponatremia was 11.8% in patients with AF without HF, which was lower than before. After multivariable adjustment, the hyponatremia remained associated with decreased survival. This could be because of lower serum sodium causing decreased Na^+^ influx, reduced transmembrane potential, inhibition of Na^+^/K^+^ ATPase activity (30), or more triggered electrical activity and burst in the pulmonary vein (31). The main interactions among the three involve neurohormone-induced hyponatremia, renin-angiotensin-aldosterone system activation, retention of water and sodium, links to fluid overload, and atrial myocardial stretch facilitation of AF (32). Our data supplement and emphasize the prognostic importance of hyponatremia at admission in patients with AF without HF.

We also evaluated the association between hypernatremia and post-discharge mortality. Our study suggests that hypernatremia at the time of hospital admission is highly related to risk of adjusted all-cause mortality within 365 days after discharge for AF patients without HF. Studies have reported that hypernatremia indicates adverse prognosis for the medical patients (14, 33–35). Breen et al. showed the same result in a cardiac intensive care unit in a retrospective study (14). A single-center cohort study that included 55,901 patients has shown that the impact on higher 1-year mortality is more prominent for hypernatremia than for normonatremia (18). The relationship between hypernatremia and adverse prognosis could be due to decreased left ventricular contractility (36) and/or increased peripheral insulin resistance (37). A case report showed that three hypernatremic patients incurred AF during treatment for hypernatremia (38). It is possible that hypernatremia could be a risk factor for AF-related mortality because of atrial stretch during treatment-stimulated pulmonary vein electrical activity (39). The reason why hypernatremia is a risk factor for patients with AF may be associated with treatment of hypernatremia.

## Limitations

First, our study is a single-center one and our participants live in the south of China, which might limit the generalizability of our results to other populations. Second, we only included the serum sodium concentration at the time of admission and did not identify the changes in the hospital. Third, we did not evaluate the association between dysnatremia and cardiac-related mortality. In future studies, we will establish the relationship between changes in serum sodium concentration and cardiac-related mortality of patients with AF.

## Conclusion

In conclusion, hypo- and hypernatremia increase the mortality of patients with AF without HF. Sodium concentrations need to be monitored after discharge from the hospital, especially in patients who have dysnatremia at the time of hospital admission.

## Data availability statement

The raw data supporting the conclusions of this article will be made available by the authors, without undue reservation.

## Ethics statement

The studies involving human participants were reviewed and approved by the Ethics Committee of the First Affiliated Hospital of Shantou University Medical College. The patients/participants provided their written informed consent to participate in this study.

## Author contributions

XT, YC, and YZ contributed to the conception and design of the study. DL, SW, JX, MY, ZX, MW, and RC organized the database. YZ and YC performed the statistical analysis. YZ wrote the first draft of the manuscript. YC, ZC, and CT wrote sections of the manuscript. All authors contributed to manuscript revision, read, and approved the submitted version.

## Funding

This study was supported by the Grant for Key Disciplinary Project of Clinical Medicine under the Guangdong High-level University Development Program, Guangdong University Innovation Team Project (Nature) (2019KCXTD003), 2020 Li Ka Shing Foundation Cross-Disciplinary Research Grant (2020LKSFG19B) and Dengfeng Project for the construction of high-level hospitals in Guangdong Province–the First Affiliated Hospital of Shantou University Medical College, the study of mechanism and early warning model of Hypertensive Disorders of Pregnancy–construction of cohort and specimen bank of pregnant women and fund of Science and Technology Special in Guangdong Province (Big Project + Task lists) (2021010303) and Transcriptome mechanisms of acute Stanford type A aortic dissection (STKJ2021083).

## Conflict of interest

The authors declare that the research was conducted in the absence of any commercial or financial relationships that could be construed as a potential conflict of interest.

## Publisher's note

All claims expressed in this article are solely those of the authors and do not necessarily represent those of their affiliated organizations, or those of the publisher, the editors and the reviewers. Any product that may be evaluated in this article, or claim that may be made by its manufacturer, is not guaranteed or endorsed by the publisher.
