# Supplementary material for: Dysnatremia is associated with increased risk of all-cause mortality within 365 days post-discharge in patients with atrial fibrillation without heart failure: A prospective cohort study
Source: Front Cardiovasc Med. 2022 Oct 12;9:963103. doi: 10.3389/fcvm.2022.963103 (PMC9597697; doi:10.3389/fcvm.2022.963103)
Supplement: Supplementary file 1 [file Table_1.docx]

**Table S1 The cause of death**

Infection including severe pneumonia and septic shock.

| **Cause of death** | **Hyponatremia**  **n = 35** | **Normonatremia**  **n = 116** | **Hypernatremia**  **n = 14** |
| --- | --- | --- | --- |
| **Known** | 1 | 8 | 1 |
| Acute heart failure | 1 | 0 | 0 |
| Cerebral infarction | 0 | 1 | 1 |
| Cerebral hemorrhage | 0 | 2 | 0 |
| Infection | 0 | 4 | 0 |
| Other reasons | 0 | 1 | 0 |

1. D. Y. Chiu, P. A. Kalra, S. Sinha and D. Green: Association of serum sodium levels with all-cause and cardiovascular mortality in chronic kidney disease: Results from a prospective observational study. *Nephrology (Carlton)*, 21(6), 476-82 (2016) doi:10.1111/nep.12634
